# Two Cases of Ruptured Intestine

**Published:** 1907-03

**Authors:** Harold F. Mole

**Affiliations:** Assistant-Surgeon and Surgeon in Charge of the Aural Department, Bristol Royal Infirmary.


### TWO CASES OF RUPTURED INTESTINE.

BY

HAROLD F. MOLE, F.R.C.S.,

*Assistant-Surgeon and Surgeon in Charge of the Aural Department, Bristol Royal Infirmary.*

As cases of rupture of the intestine do not appear to be very common, and as, at any rate, cases operated on are seldom reported, it seems to me that these two successful ones are worthy of record.

**Case 1.**—Joseph K., æt. 35, was admitted to the Bristol Royal Infirmary on the evening of July 27th, 1904, complaining of severe abdominal pain. He had been struck in the epigastric region, slightly to the left of the middle line, by the pole of a swing-boat. He was taken unawares, being quite unprepared for the blow. He walked into the Infirmary. He was an exceedingly poor man, and had had no food for eight hours. He was much collapsed immediately after the blow, and in a few minutes vomited. After a time he recovered sufficiently to walk to a tram and come to the Infirmary. On admission he was rather collapsed, his abdomen hard and tender in the upper part, pulse good, 62, and temperature 95° F. He was watched for two hours, and during this time vomited three times, the total amount, however, being only two ounces. I saw him at this time, and his pulse was 72, very good, his temperature 97° F., and his abdomen rather hard and tender in the epigastric region. There was some blood in the vomit, corpuscles being seen with the microscope. The nature of the accident and the history strongly suggested to me a rupture of the intestine, and the abdomen was opened at once, four hours after the accident, by a median incision above the umbilicus. Blood appeared on opening the peritoneum, and on displacing coils of intestine was seen to come from the neighbourhood of the spleen. There was no sign of stomach or intestinal contents except a little sticky mucus. The stomach and spleen were carefully examined, but no rupture detected. The small intestine was then pulled out from the upper abdomen, and upper part of the jejunum being exposed, a large tear was seen involving nearly half the circumference of the bowel, but being placed obliquely to its long axis, in its fixed part just beyond the duodeno-jejunal junction. The thick mucous membrane was markedly everted, giving rise to a red-looking tumour. The blood was mopped out, and the mucous membrane sewn up with a continuous catgut stitch. The peritoneal and muscular coats were brought together with interrupted Halsted's stitches, and outside these one or two supporting Lembert stitches. The suturing was a little difficult owing to the fixed position of the bowel posteriorly. The abdomen was washed out with saline, and some of this left in, and a large cigarette drain inserted down to the line of suture. The rest of the wound was sewn up. Four days later the drain was removed and replaced by a very much smaller one, which was removed next day. He was allowed only water and brandy by the mouth for the first few days, and was given nutrient enemata. Other fluids were soon added, but no actual solid food until the fourteenth day, when the stitches were removed. He made an uninterrupted recovery, and never thought there was much the matter with him; consequently, we had great difficulty in keeping him in bed and making him submit to the restricted diet. He was discharged on the twentyeighth day, and seen about a year later was remaining quite well.

**Case 2.**—Charles J., æt. 16, was admitted to the Bristol Royal Infirmary on the morning of September 17th, 1906. He was working in a fitting shop when the arm of a crane swung round and struck him in the abdomen, pinning him against some railway sleepers. He was set free by the wood being sawn away from behind him. He then, to use his own expression, got up, put his hands in his pockets and walked to the office. On admission he appeared to be somewhat concussed (there was a scalp wound), and the abdominal symptoms masked; he was somewhat pale, pulse 84, and temperature 97° F. Abdominal rigidity soon appeared, and rapidly became more marked. He also became blanched, and in two hours his pulse rose from 84 to 120 per minute. When I saw him, about four hours after the accident, he was clearly suffering from internal hemorrhage. Pallor was extreme, pulse 136, restless, and much abdominal pain and tenderness, with rigidity most marked under the upper part of the left rectus. I made a probable diagnosis of rupture of the left lobe of liver or spleen, and would have operated at once, but had to wait nearly two hours to obtain the consent of the parents. Six hours after the accident I made an incision in the middle line above the umbilicus, and rapidly examined the liver and spleen, and found them normal. From amidst a large mass of blood-clot to the left I delivered a piece of intestine, jejunum I judged by its thickness, completely torn through transversely in its whole circumference, and including two to three inches of its mesentery. It might almost have been done with a pair of scissors, except that the mucous membrane was somewhat jagged. The mucous membrane was everted, and what I took to be the upper end of the bowel was filled with blood-clot. There was an artery spurting in the mesentery. There was no sign of extravasation of intestinal contents. I judged that the injury had probably been caused by the crane tearing the bowel across the spine. The bleeding vessel was ligatured, the mesentery sewn up on either side with sutures applied after Lembert's method with silk. A continuous stitch brought the mucous membrane together, and the peritoneo-muscular coats were united with continuous Lembert sutures starting on either side at the end of the mesenteric sutures and being united in the middle line. As the bowel could be delivered outside the parietes, the suturing was easy, and was performed rapidly, as the condition of the patient was precarious. No attempt was made to swab or wash out the abdomen or to remove the blood-clot. Saline fluid was run into the abdomen whilst the wound was being sewn up, and no drainage was made. For the remainder of the day the boy was extremely bad, and had rectal injections of saline, and saline infusions into the veins, nutrient enemata, and only water and brandy by the mouth. His temperature rose to 100° F. the night of the operation, but there was never fever after this. For two days his condition gave rise to some anxiety from abdominal distension and rapid pulse, but after this his recovery was interrupted. He ate chicken on the ninth day, the stitches were removed on the fourteenth, and he was discharged at the end of a month. I saw him two months after, and he seemed quite well.

In addition to these two cases, I have distinct recollection of three others under the care of my colleagues during the past five or six years, two of which recovered and one died. The latter was extremely bad at the time of operation, and his abdomen was found full of fæces. I am therefore led to suppose that traumatic ruptures of the intestine uncomplicated by other serious abdominal injuries give very favourable results when operated on early. It is therefore of the first importance to make an early diagnosis. This is by no means always easy, for the symptoms may be very equivocal, and cases are on record where patients have walked long distances after this accident. I am disposed to lay considerable stress on the nature of the accident. This will be found most usually to be a sudden, well-localised blow. Of causes that have actually come under my own observation may be mentioned the following :—A kick from a horse, coming in contact with the point of a shaft of a cart whilst riding a bicycle, falling on to the handle of a pick, and being struck by the point of a pole (Case I.). There is usually initial collapse, and frequent vomiting is said to be an important symptom. When it is present I think it is so, but I don't know that I should feel inclined to lay a great deal of stress on its absence. My second case had none. The one symptom which I think is, perhaps, of more value than any other, following on such an injury as I have suggested, is rigidity of the abdomen. This may be marked or only slight, and limited to the area struck, but it is none the less of great importance. There were some who thought there was hardly sufficient indication to operate in my first case, but the nature of the accident, plus the rigidity, decided me. There was also frequent vomiting, considering the length of time that elapsed and the absence of food from the stomach. The pulse, however, was no assistance whatever, and this is not infrequently the case, whilst in another class of abdominal conditions, notably appendicitis, it may be the best guiding sign of the serious condition of the patient. I was fortunate in these two cases in that there was no obvious extravasation of fæces in either. This was accounted for to some extent by the bowel being more or less empty, but also by the way the thick mucous membrane of the jejunum becomes everted, almost completely blocking quite a large hole. Blood-clot also helps to block the opening. My second case was of interest in that the symptoms of internal hemorrhage quite masked those of intestinal rupture, and thus made it a complicated case. The blood came from torn vessels in the mesentery. It would appear that in cases of this kind, without visible fæces in the peritoneum, it is quite safe to sew up the abdomen without drain, as is shown by my second case, and I have little doubt my first would have done equally well, but I felt a little uncertain about the suturing as it was difficult. It is also clear that it does no harm to leave a large mass of blood-clot in the abdomen. With regard to the vexed question of flushing the abdomen, I would suggest that this procedure be limited to those cases in which there is wide diffusion of fæces, and that in the other cases the locally infected area be carefully swabbed dry, with as little disturbance of the neighbouring parts as possible. Treves says, in his *System of Surgery* (1896), that the jejunum is the most common seat of rupture (two of the three other cases referred to were ileum, and the third I do not remember), that in only 16 per cent. of all cases is the rupture complete, that in about 70 per cent. there is escape of fæces into the peritoneal cavity, and that in about 15 per cent. there is a rent in the mesentery. The following results of operation are given : 24 cases of incomplete rupture with 11 deaths, and 4 cases of complete rupture with 3 deaths.

